# Apocrine encapsulated papillary carcinoma of the breast with microscopic multifocal capsular invasion: a case report

**DOI:** 10.3389/fonc.2026.1895275

**Published:** 2026-07-07

**Authors:** Jiang Wang, Minjun Lu, Lijing Jiang

**Affiliations:** 1 Department of Thyroid and Breast Surgery, Tianxiang Medical Dongfang Hospital, Yiwu, China; 2 Department of Thyroid and Breast Surgery, Dongyang Hospital of Wenzhou Medical University, Dongyang, China; 3 Department of Pathology, Dongyang Hospital of Wenzhou Medical University, Dongyang, China

**Keywords:** androgen receptor, apocrine differentiation, encapsulated papillary carcinoma, microscopic multifocal invasion, PIK3CA mutation

## Abstract

**Background:**

Apocrine encapsulated papillary carcinoma (A-EPC) of the breast is exceedingly rare, having been described primarily through sporadic case reports prior to 2022 (typically fewer than 10 cumulative cases per report). The largest series to date — 28 cases from a single centre — was recently published in 2026. Whether breast-conserving surgery (BCS) is appropriate for A-EPC with frank invasion, and how the recent Rakha–Quinn (2025) staging criteria should be applied, remain unresolved.

**Case presentation:**

A 57-year-old postmenopausal woman presented with a 5-month history of an enlarging right breast mass. Imaging demonstrated a 27 × 17 × 17 mm well-circumscribed nodule at the 9 o’clock position, approximately 2 cm lateral to the nipple. Intraoperative frozen section identified prominent apocrine cytological features, predicting a hormone-receptor-negative phenotype; same-session sentinel lymph node biopsy (SLNB) was therefore performed during the index operation. Successful BCS with negative margins was achieved. Final histopathology demonstrated A-EPC (2.3 × 1.8 × 1.5 cm) with microscopic multifocal frank invasion (largest focus 4 mm; pT1aN0M0; 0/4 sentinel nodes). Immunohistochemistry confirmed ER−/PR−/HER2 2+ (FISH-negative)/AR+ (~90%)/GCDFP-15+/Ki-67 ~15%. A PIK3CA exon 20 H1047R/L hotspot mutation was detected. After multidisciplinary discussion, no systemic therapy was administered and the patient completed adjuvant whole-breast radiotherapy.

**Conclusions:**

Three points were central to this case: intraoperative frozen-section recognition of apocrine differentiation supported same-session SLNB during breast-conserving surgery; systematic application of the Rakha–Quinn (2025) staging criteria — including the 5 mm proximity rule and the assessment of whether microscopic multifocal invasion indicates a diffusely invasive EPC-like pattern — supported staging as pT1a; and the co-occurrence of strong androgen-receptor positivity and a PIK3CA mutation provides a rationale for AR-PI3K dual-pathway targeted therapy should recurrence occur.

## 1 Introduction

Encapsulated papillary carcinoma (EPC) is a rare malignancy accounting for 0.5–2% of all breast carcinomas (1, 2). It typically affects postmenopausal women, presents as a centrally located mass with low-to-intermediate nuclear grade, and is most often hormone-receptor-positive with a luminal A-like profile. The apocrine variant (A-EPC) is exceedingly rare, having been described primarily through sporadic case reports prior to 2022 (typically fewer than 10 cumulative cases at the time of each report), with the largest series to date — 28 cases from a single centre — only recently published by Zuo et al. in 2026 (3). A-EPC is morphologically defined by ≥90% apocrine differentiation and characterised by a triple-negative immunophenotype (ER−/PR−/HER2−) with strong androgen receptor (AR) and GCDFP-15 expression (4).

Two questions of practical importance arise when A-EPC is encountered. First, when is sentinel lymph node biopsy (SLNB) indicated in this rare variant? Classical EPC is most often pure *in-situ* disease (pTis), and even when accompanied by a small invasive component is typically hormone-receptor-positive and HER2-negative — a profile that increasingly aligns with the SLNB-omission populations established by the SOUND (5) and INSEMA (6) randomised trials and incorporated into the 2025 ASCO guideline update on SLNB in early-stage breast cancer (7). A-EPC differs fundamentally: published cases have shown a high rate of accompanying frank invasion (3, 8, 9), and the triple-negative immunophenotype removes A-EPC from these de-escalation pathways regardless of whether invasion can be confirmed at the time of preoperative core biopsy or intraoperative frozen section. The diagnosis of A-EPC itself — established by recognition of apocrine differentiation in a circumscribed papillary lesion — therefore provides sufficient grounds for SLNB, without waiting for histological confirmation of invasion on permanent sections. Second, how should true frank capsular invasion be evaluated and staged in this rare entity, especially when invasion is microscopic multifocal? The Rakha–Quinn (2025) framework has provided explicit criteria — including the 5 mm proximity rule and the assessment of microscopic multifocal invasion in determining whether the whole tumour represents an EPC-like invasive carcinoma — but these have not yet been illustrated in published A-EPC cases (2).

We present a case of A-EPC with multifocal frank capsular invasion. We describe the surgical decision-making process, apply the Rakha–Quinn (2025) framework to the histopathological findings, and discuss the molecular profile in the context of recently identified actionable targets.

## 2 Case presentation

### 2.1 Clinical history and physical examination

A 57-year-old postmenopausal woman presented with a 5-month history of a palpable mass in the right breast, which she perceived to have gradually enlarged over the preceding months. On retrospective inquiry, she recalled subjectively that breastfeeding many years earlier had been less productive on the right side compared to the left — an observation of unclear clinical significance. There was no nipple discharge, skin retraction, or family history of breast or ovarian malignancy.

Physical examination revealed a mobile, well-circumscribed mass in the right breast, located approximately 2 cm lateral to the nipple. The overlying skin and nipple-areolar complex were unremarkable. Axillary lymph nodes were not palpable on bilateral clinical examination.

### 2.2 Imaging evaluation

**Figure 1:**
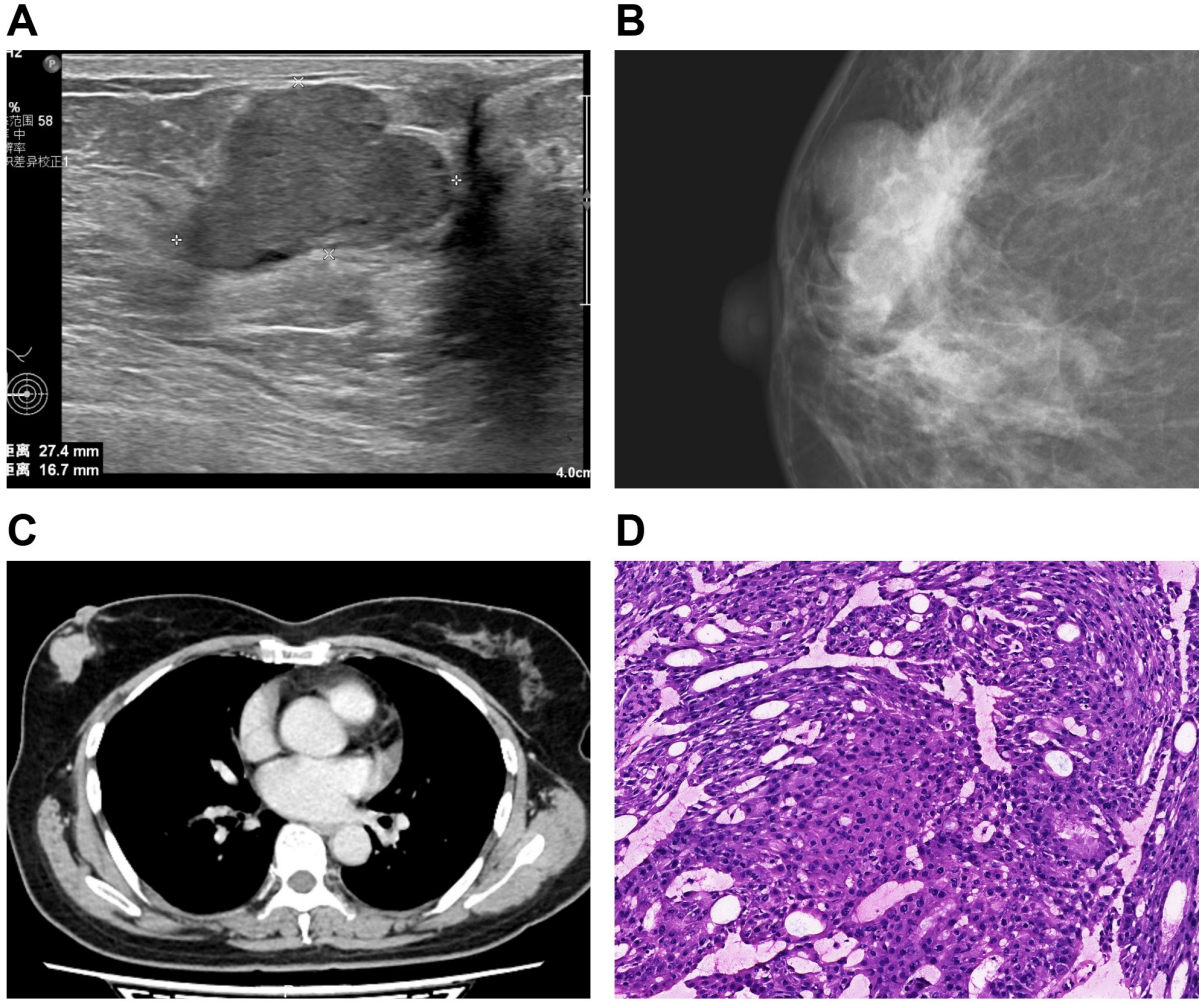
**(A)** Greyscale and colour Doppler ultrasound demonstrating a well-circumscribed, lobulated hypoechoic nodule measuring 27 × 17 × 17 mm at the 9 o’clock position of the right breast, with smooth margins, heterogeneous internal echogenicity, no calcifications, and abundant intralesional vascularity. **(B)** Mammography (right craniocaudal view) showing a circumscribed, dense, lobulated mass in the lateral aspect of the right breast with smooth margins, no microcalcifications, and a preserved distance between the mass and the nipple-areolar complex. **(C)** Contrast-enhanced chest CT (axial view) confirming the well-defined mass lateral to the nipple, with a clear boundary against the surrounding fibroglandular tissue and no chest-wall involvement or pathological adenopathy. **(D)** Intraoperative frozen section (H&E) revealing a circumscribed papillary lesion with prominent apocrine cytological features — abundant eosinophilic cytoplasm, prominent nucleoli, and apical snouts — prompting same-session sentinel lymph node biopsy.

Bilateral breast ultrasound demonstrated a well-circumscribed hypoechoic nodule measuring 27 × 17 × 17 mm at the 9 o’clock position of the right breast (**Figure 1A**). The lesion showed coarse lobulation, smooth margins, heterogeneous internal echogenicity, no calcifications, and abundant intralesional vascularity on colour Doppler imaging. Bilateral axillary nodes appeared morphologically benign on ultrasound — oval-shaped with preserved fatty hila — concordant with the negative clinical axillary examination.

Mammography (right craniocaudal view; **Figure 1B**) demonstrated a circumscribed, dense, lobulated mass in the lateral aspect of the right breast with smooth margins and no microcalcifications. A distinct distance between the mass and the nipple-areolar complex was preserved, supporting the feasibility of conservative resection. Contrast-enhanced chest CT (**Figure 1C**) confirmed the lesion’s location lateral to the nipple, demonstrated a well-defined boundary against the surrounding fibroglandular tissue, and excluded chest-wall invasion or pathological adenopathy.

### 2.3 Surgical management and intraoperative decision-making

Preoperative ultrasound, mammography, and CT consistently showed a well-circumscribed solid mass with smooth margins, for which the probability of a benign lesion was not low, and as the need for neoadjuvant down-staging to permit breast conservation was not strong in this patient, preoperative core needle biopsy was not considered essential. After thorough discussion with the patient, excisional surgery with intraoperative frozen-section assessment was chosen to achieve diagnosis and definitive treatment in a single procedure.

Given the clinical and radiological features — a well-circumscribed mass of modest size with adequate distance from the nipple — BCS was planned. The patient underwent right breast-conserving wide local excision with intraoperative frozen section, which revealed a circumscribed papillary lesion with prominent apocrine cytological features — abundant eosinophilic cytoplasm, prominent nucleoli, and apical snouts (**Figure 1D**) — raising the possibility of A-EPC. The limited resolution of frozen sections precluded definitive exclusion of stromal invasion, and immunohistochemical receptor status could not be assessed intraoperatively. On the basis of the apocrine morphology recognised intraoperatively, same-session sentinel lymph node biopsy was performed to obtain accurate axillary staging in the event that a chemotherapy decision would be required postoperatively.

Following the intraoperative frozen-section diagnosis, sentinel lymph node biopsy was performed using a dual-dye method (carbon nanoparticles followed by methylene blue, both injected at several subareolar subcutaneous points). Stained lymphatic channels were traced to the draining nodes, and both blue- and black-stained nodes were excised as sentinel lymph nodes; no unstained but clinically suspicious nodes were encountered.

### 2.4 Gross and microscopic pathology

**Figure 2:**
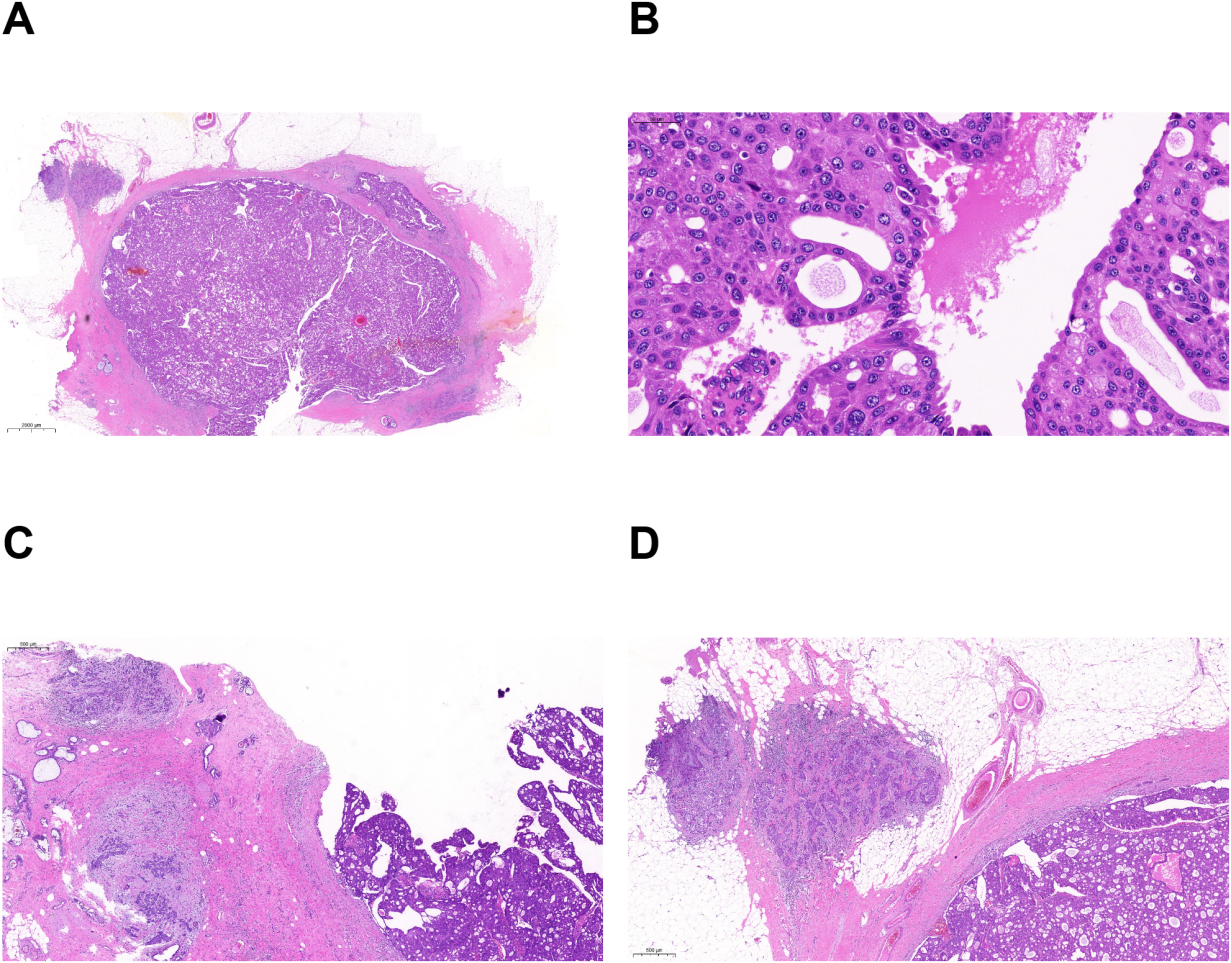
**(A)** Low-power view (H&E) demonstrating the overall architecture with a well-defined fibrous pseudo-capsule. **(B)** High-power view (H&E) showing characteristic apocrine cytology with abundant eosinophilic granular cytoplasm, central round nuclei, prominent nucleoli, and apical snouts. **(C)** Medium-power view (H&E) showing multiple foci of frank invasion within fibroadipose tissue beyond the fibrous capsule. **(D)** Higher-power view of a representative invasive focus (H&E), confirming neoplastic epithelial nests permeating the periductal stroma. The largest invasive focus measured 4 mm and was located approximately 0.5 mm beyond the capsule.

The wide local excision specimen contained an encapsulated mass measuring 2.3 × 1.8 × 1.5 cm. Histologically, the lesion demonstrated a papillary and cribriform proliferation surrounded by a thick fibrous pseudo-capsule (**Figure 2A**). At higher magnification, the neoplastic epithelium displayed unequivocal apocrine differentiation in >90% of cells: large polygonal cells with abundant eosinophilic granular cytoplasm, central round nuclei, prominent nucleoli, and visible apical snouts (**Figure 2B**). Nuclear atypia was mild to moderate; mitotic activity was scant. Stromal tumour-infiltrating lymphocytes (TILs) were estimated at approximately 10%.

**Figure 3:**
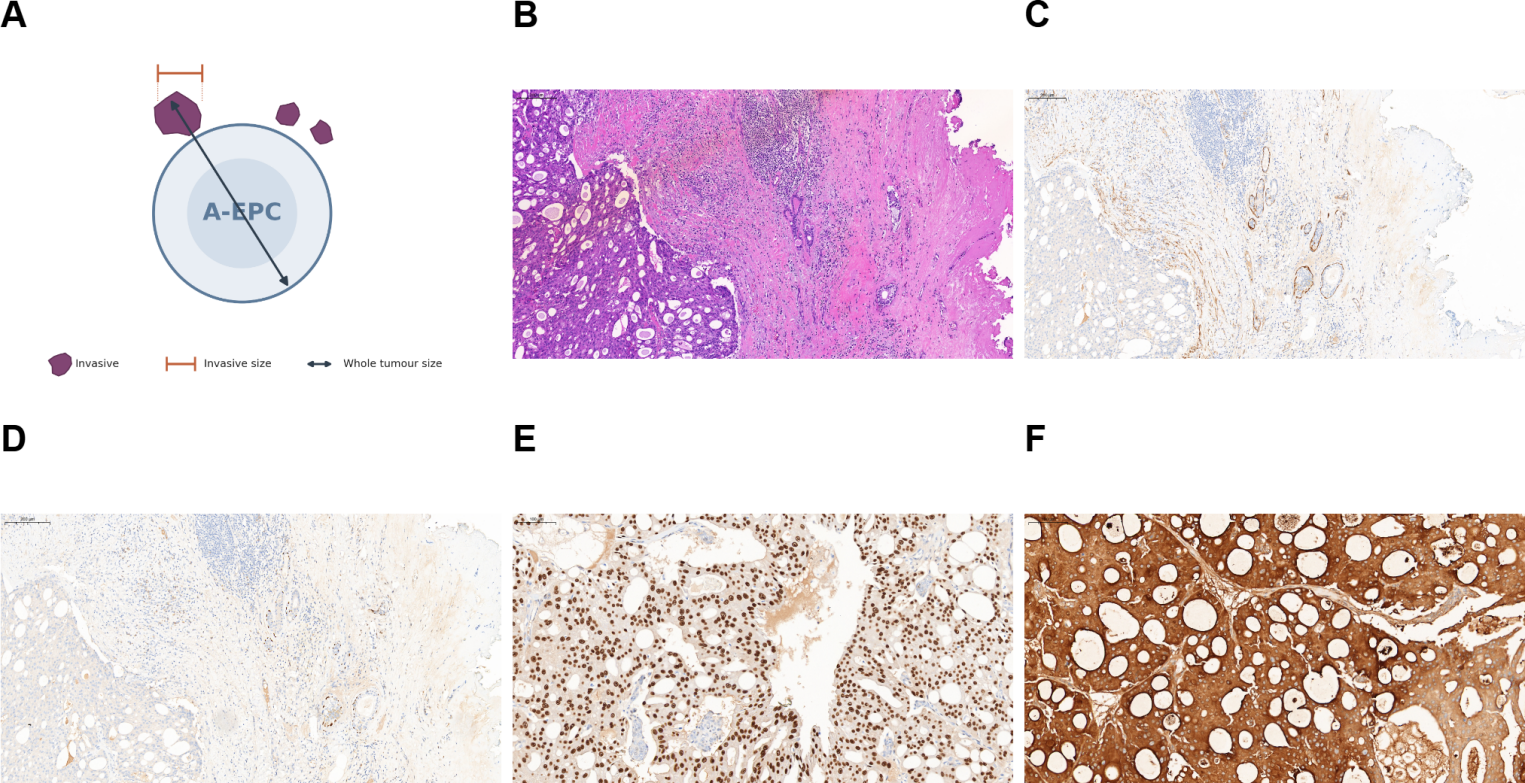
**(A)** Schematic representation of the spatial distribution of invasive foci reconstructed from serial sections, corresponding to the first scenario illustrated in Figure 9 of Rakha and Quinn (2025) (2) — invasive foci (blue spiked elements) are confined to one sector of the tumour periphery rather than distributed extensively around the entire EPC (central orange area with red border), a pattern consistent with staging by the largest invasive focus rather than by whole tumour size. The red bar represents the size scale of the largest invasive focus (Invasive size); the black diagonal line represents the whole tumour size. **(B)** Medium-power view (H&E) showing tumour (left) and adjacent normal breast ducts (right). **(C)** Calponin immunohistochemistry demonstrating absence of myoepithelial cells throughout the tumour while preserving a continuous myoepithelial layer in adjacent normal ducts (right), serving as a positive internal control. **(D)** p63 immunohistochemistry showing the same pattern — complete loss of myoepithelial nuclear staining in the tumour and intact myoepithelial expression in adjacent normal ducts. **(E)** Androgen receptor (AR) showing strong, diffuse nuclear positivity in approximately 90% of tumour cells. **(F)** GCDFP-15 showing strong cytoplasmic positivity consistent with apocrine differentiation.

Multiple foci of frank invasion were identified within the fibroadipose tissue beyond the fibrous capsule, demonstrating an irregular, cord-like infiltrative growth pattern with associated stromal response (**Figures 2C, D**). The largest invasive focus measured 4 mm and was located approximately 0.5 mm beyond the EPC capsule (**Figure 2D**). The sampled invasive foci appeared confined to one region of the tumour periphery rather than encircling the lesion; the three-dimensional distribution was reconstructed from review of serial sections (**Figure 3A**). No lymphovascular invasion or perineural invasion was identified. All resection margins were negative. Four sentinel lymph nodes were examined and all were negative.

Comparison of tumour with adjacent normal breast tissue (**Figure 3B**) clearly highlighted architectural and cytological differences. Immunohistochemistry for myoepithelial markers — calponin (**Figure 3C**) and p63 (**Figure 3D**) — demonstrated complete absence of myoepithelial cells throughout the tumour and around the invasive foci, while normal ducts in the adjacent parenchyma served as positive internal controls.

### 2.5 Immunohistochemistry and molecular findings

Tumour cells were diffusely and strongly positive for AR (~90%; **Figure 3E**) and GCDFP-15 (**Figure 3F**), confirming apocrine differentiation. ER and PR were negative. HER2 immunohistochemistry showed equivocal 2+ membranous staining, prompting reflex fluorescence *in-situ* hybridisation (FISH), which demonstrated a HER2/CEP17 ratio of 1.3 and average HER2 copy number of 2.0 — HER2 not amplified. Ki-67 was approximately 15%. PD-L1 combined positive score (CPS) was approximately 5. p53 staining showed a wild-type pattern, and E-cadherin was retained. Targeted PIK3CA hotspot mutation testing by PCR detected an exon 20 (H1047R/L) mutation, whereas exon 9 hotspot mutations (E542K and E545K/D) were not identified.

### 2.6 Final diagnosis

Apocrine encapsulated papillary carcinoma of the right breast with microscopic multifocal frank invasion (largest invasive focus 4 mm; invasive foci confined to one sector of the tumour periphery on serial-section review, without evidence of encircling the entire lesion); ER−/PR−/HER2 2+ (FISH non-amplified)/AR+ (90%, strong)/GCDFP-15+/Ki-67 ~15%/PD-L1 CPS≈5; PIK3CA exon 20 H1047R/L mutation. pT1aN0 (0/4 sentinel nodes) M0. The application of the Rakha–Quinn (2025) diagnostic and staging criteria to the case is summarised in **Table 1**.

**Table 1:** Application of Rakha–Quinn (2025) diagnostic and staging criteria to the present case.

Diagnostic criterion	Rakha–quinn 2025 rule	Findings in present case	Implication
True frank invasion vs intracapsular entrapment	Irregular infiltration beyond capsule with stromal response = true invasion; entrapped fat cells = definitive sign	Microscopic multifocal cord-like invasion in fibroadipose tissue beyond capsule with stromal response	Classified as true frank invasion
Proximity rule	≤5 mm from EPC = arises from EPC; >5 mm = separate focus	All invasive foci ~0.5 mm from EPC capsule	Invasion arises from EPC; single tumour staging
Spatial distribution of microscopic multifocal invasion	Multiple foci at the EPC periphery suggest the whole tumour represents invasive carcinoma with an EPC-like pattern (2)	Invasive foci confined to one sector of the tumour periphery without encircling the lesion	Invasive foci do not indicate a wholly invasive EPC-like pattern; staged by largest invasive focus
Tumour size for T staging	Largest invasive focus = invasive size; whole lesion = overall tumour size	Largest invasive focus 4 mm; overall lesion 2.3 cm	pT1a (invasive component ≤5 mm)
Biomarker assessment	Performed on the invasive component	Invasive foci show same A-EPC immunoprofile (ER−/PR−/HER2−/AR+)	Treatment decisions based on invasive component
SLNB recommendation	Recommended when invasion is identified	SLNB performed at index operation; 0/4 positive	pN0; confirms low-risk axillary status

A-EPC, apocrine encapsulated papillary carcinoma; AR, androgen receptor; EPC, encapsulated papillary carcinoma; ER, oestrogen receptor; HER2, human epidermal growth factor receptor 2; PR, progesterone receptor; pT, pathological tumour stage; pN, pathological nodal stage; SLN, sentinel lymph node; SLNB, sentinel lymph node biopsy.

### 2.7 Adjuvant treatment, multidisciplinary discussion, and follow-up

The case was discussed at the institutional multidisciplinary tumour (MDT) board. The invasive component was small (largest focus 4 mm; pT1a) and node-negative (0/4 sentinel nodes), and the tumour showed strong AR positivity (~90%) with a low-to-moderate Ki-67 (~15%). On this basis, the MDT considered the patient to be at low risk of recurrence.

After detailed counselling, the patient declined chemotherapy. As ER and PR were negative, endocrine therapy was not indicated. Anti-HER2 therapy was not indicated given the FISH-negative result. Approximately one month after surgery, the patient commenced adjuvant radiotherapy to the right breast, which was completed without complications.

At the time of manuscript submission, the patient had completed three months of postoperative follow-up. The surgical sites of the right breast and axilla had healed well, with no palpable lymphadenopathy, and local ultrasonography showed no evidence of recurrence.

### 2.8 Patient perspective

The patient expressed initial anxiety regarding the unfamiliar diagnosis and the apparent contradiction between the small invasive component and the triple-negative phenotype. After multidisciplinary discussion and detailed counselling, she made an informed decision to forgo chemotherapy. She expressed satisfaction with the breast-conserving outcome and provided written informed consent for publication of this case report and all accompanying images.

## 3 Discussion

### 3.1 Morphological features and clinical behaviour of the apocrine variant

EPC is a well-circumscribed papillary lesion typically presenting in postmenopausal women, characterised by anastomosing fibrovascular cores and a fibrous pseudo-capsule (1, 2). The apocrine variant is morphologically defined by ≥90% apocrine differentiation and characterised by a triple-negative phenotype with strong AR and GCDFP-15 expression (4, 10). Until recently, A-EPC had been described almost exclusively through sporadic case reports, with individual publications typically referencing fewer than 10 cumulative cases. The 28-case series by Zuo et al. (2026) confirmed that A-EPC tends to display higher nuclear grade than classical EPC, yet its overall biological behaviour remains closer to that of classical EPC than to conventional TNBC (3).

### 3.2 Intraoperative rationale for axillary staging in the era of de-escalation

The recognition of apocrine cytology on intraoperative frozen section — abundant eosinophilic cytoplasm, prominent nucleoli, and apical snouts — provides diagnostic information equivalent in scope to what would be available from a preoperative core needle biopsy. Although neither modality can reliably establish or exclude an invasive component, owing to limited tissue sampling and the inability to perform myoepithelial immunohistochemistry intraoperatively, both can establish the diagnostic possibility of A-EPC based on apocrine morphology in a circumscribed papillary lesion. Because apocrine cells are biologically programmed to be hormone-receptor-negative and AR-positive, intraoperative identification of apocrine morphology effectively predicts a triple-negative immunophenotype.

The diagnostic possibility of A-EPC alone — independent of whether invasion has been histologically confirmed — has immediate implications for axillary surgery. Classical EPC is most often pure *in-situ* disease, with reported SLN positivity rates below 3% even when small invasive components are present (11–13), and any invasive component is typically hormone-receptor-positive and HER2-negative. The SOUND randomised trial demonstrated non-inferiority of SLNB omission in patients with tumours ≤2 cm, negative axillary ultrasound, and predominantly luminal biology (5); the INSEMA trial extended this finding to a broader cohort undergoing breast-conserving therapy (6); and the 2025 ASCO guideline update formally endorses SLNB omission in selected postmenopausal patients with cT1N0, hormone-receptor-positive, HER2-negative breast cancer undergoing breast-conserving surgery with adjuvant radiotherapy and endocrine therapy (7). A-EPC patients do not fit these criteria on two independent grounds. First, the triple-negative immunophenotype excludes them from current de-escalation pathways. Second, published A-EPC case series and reports have demonstrated a high rate of accompanying frank invasion (3, 8, 9), which substantially elevates the prior probability of nodal involvement even when invasion has not yet been confirmed on permanent sections. Once frozen section raises the possibility of A-EPC, same-session SLNB at the index operation therefore becomes the safer default, sparing the patient a second axillary procedure should subsequent histological assessment confirm invasion. In our patient, this reasoning supported the decision to perform SLNB during the initial procedure, which ultimately yielded 0/4 negative nodes.

### 3.3 Distinguishing true capsular invasion: principles and application

The Rakha–Quinn (2025) framework defines true frank invasion as neoplastic elements that permeate beyond the fibrous capsule with an irregular infiltrative pattern, typically accompanied by a desmoplastic stromal response; the presence of entrapped fat cells is regarded as a definitive sign (2). Diagnostic mimics include intracapsular epithelial entrapment within zones of reactive fibrosis and biopsy-site epithelial displacement. In our case, the cord-like infiltration into fibroadipose tissue with associated stromal response made the distinction straightforward.

More clinically consequential was the 5 mm proximity rule: an invasive focus ≤5 mm from the EPC is considered to arise from the EPC and is staged as part of the same lesion, whereas a focus >5 mm away is treated as a separate microscopic multifocal carcinoma (2). In our patient, all invasive foci were located approximately 0.5 mm from the capsule — well within the threshold — supporting an integrated diagnosis of “A-EPC with associated frank invasion” rather than synchronous independent tumours.

### 3.4 Tumour size assessment in microscopic multifocal invasion versus true macroscopic multifocality

A prerequisite for size assessment is clarifying what “multifocal” means in this context. The term “multifocal” in the present report refers specifically to microscopic multifocal capsular invasion arising from a single primary lesion, and should not be conflated with true macroscopic multifocal breast cancer. By conventional definition, true multifocality requires two or more spatially distinct macroscopic invasive foci separated by intervening normal breast tissue (14). Because the entire encapsulated lesion in our patient measured only 2.3 cm, multiple macroscopic foci separated by such distances were not present; the invasive foci instead clustered immediately beyond a single capsule. Accordingly, the tumour was staged on the basis of the single largest invasive focus (4 mm; pT1a) rather than as an aggregate or diffusely invasive lesion.

Rakha and Quinn (2025) noted that accurate assessment of invasive tumour size may be challenging in EPCs with more than one focus of accompanying invasive carcinoma, particularly if these are separately located at the periphery of the tumour rather than forming a discrete invasive component (2). Their framework provides a visual guide to measurement (Figure 9 in reference 2), contrasting two scenarios: in the first, a small number of invasive foci are confined to one area of the EPC periphery, and staging is based on the largest invasive focus alone; in the second, invasive foci are distributed so extensively around the EPC periphery as to suggest that the entire lesion represents invasive carcinoma with an EPC-like pattern, in which case the whole tumour size is used for staging. No quantitative threshold is defined — the distinction is based on an overall morphological assessment of whether the distribution of invasion implies a diffusely invasive lesion (2).

In our case, the invasive foci were confined to one sector of the tumour periphery, consistent with the first scenario illustrated in Figure 9 of Rakha and Quinn (2). The spatial distribution was reconstructed from review of serial sections and is represented schematically in **Figure 3A** of the present report. The foci did not approximate the pattern of extensive peripheral encirclement that would suggest a wholly invasive EPC-like carcinoma. The lesion was therefore staged based on the largest invasive focus (4 mm; pT1a). Had the entire lesion been classified as invasive by whole tumour size, the pT stage would have been 2.3 cm (pT2), with materially stronger arguments for adjuvant chemotherapy, illustrating the practical importance of this assessment.

### 3.5 Prognostic considerations, management, and follow-up

The small invasive component, node-negative status, and favourable biomarker profile in our patient support an optimistic outlook, and the conclusion of Zuo et al. (2026) that A-EPC behaves biologically closer to classical EPC than to conventional TNBC further favours a measured management approach (3). Within this low-risk context, breast-conserving surgery was both appropriate and feasible: the modest overall tumour size, a location approximately 2 cm lateral to the nipple with a well-circumscribed boundary, and a small invasive component confined to one sector of the tumour periphery together permitted wide excision with negative margins, consistent with population-based data showing equivalent overall survival and low local recurrence rates for breast conservation plus radiotherapy in EPC (15). Nevertheless, a uniformly indolent course cannot be assumed: even in node-negative, low-stage disease, favourable clinicopathological features do not entirely preclude systemic progression. Bouhani et al. (2025) reported a pure EPC that developed distant metastasis to the sternum and liver three months after surgery (16); although that case differed substantially from ours in tumour burden (a 13 cm mass with close margins) and patient age, it serves as a reminder that adequate local control does not fully eliminate the risk of systemic recurrence. Accordingly, while no systemic therapy was administered, structured long-term oncological surveillance is warranted; the patient remains under regular follow-up and has shown no evidence of recurrence to date.

### 3.6 Molecular landscape and actionable dual-pathway targets

The molecular profile of our patient — strong AR expression (~90%) and an activating PIK3CA exon 20 H1047R mutation — is characteristic of the molecular apocrine/LAR subtype. PIK3CA mutations are well-documented in EPC overall, with Liu et al. (2021) reporting a 46.3% mutation rate and H1047R/L as the most common hotspot (17). The recent landmark study by Nakamura et al. (2026) demonstrated PIK3CA mutations in approximately 68% of apocrine carcinomas — the highest frequency among all breast cancer subtypes analysed — with H1047R particularly enriched (18). The detection of PIK3CA H1047R in our patient is therefore consistent with both reference points and extends the molecular apocrine signature to the encapsulated papillary variant.

Although our patient does not currently require systemic therapy, this profile defines a clear path for management should recurrence occur. AR antagonists including bicalutamide, enzalutamide, and darolutamide have shown clinical activity in AR-positive triple-negative breast cancer (19). The next-generation α-selective PI3K inhibitor inavolisib demonstrated both progression-free and overall survival benefit in PIK3CA-mutated advanced breast cancer in the INAVO120 phase III trial (20, 21), although currently approved for HR+ disease. Most relevant to our patient, the TBCRC 032 trial of enzalutamide combined with the PI3K inhibitor taselisib in AR+ metastatic TNBC provided early evidence that PIK3CA-mutant LAR tumours may benefit from AR-PI3K dual-pathway targeting (22). Our patient’s profile — AR-positive at 90% and PIK3CA H1047R-mutant — corresponds closely to this strategy’s target population.

### 3.7 Limitations

This single case report cannot establish the prevalence of PIK3CA mutations in A-EPC, and the targeted PCR approach did not provide comprehensive genomic characterisation. The spatial distribution of microscopic multifocal invasion was inferred from serial sections rather than demonstrated on any single slide, a limitation inherent to assessing three-dimensional tumour geometry in routine histological practice. Longer follow-up will be required to confirm the safety of omitting systemic therapy in this setting.

## 4 Conclusion

This report describes a rare case of apocrine encapsulated papillary carcinoma with multifocal frank capsular invasion (pT1aN0) in a 57-year-old postmenopausal woman. Key clinical lessons include: (1) intraoperative recognition of apocrine differentiation provides a real-time signal of a likely triple-negative phenotype and should generally prompt same-session SLNB, avoiding a second axillary procedure once the triple-negative invasive phenotype is confirmed on permanent sections; (2) systematic application of the Rakha–Quinn (2025) criteria — including the 5 mm proximity rule and the assessment of whether microscopic multifocal peripheral invasion indicates a wholly invasive EPC-like pattern — supports staging A-EPC with limited microscopic multifocal invasion as pT1a rather than as a diffusely invasive EPC-like carcinoma (which would otherwise be staged as pT2 with stronger arguments for adjuvant chemotherapy); and (3) the co-occurrence of strong AR positivity and PIK3CA H1047R establishes a rationale for AR-PI3K dual-pathway targeted therapy in the event of recurrence or progression.

## Data Availability

The datasets presented in this study can be found in online repositories. The names of the repository/repositories and accession number(s) can be found in the article/**Supplementary Material**.
