# Supplementary figures and images for: Apocrine encapsulated papillary carcinoma of the breast with microscopic multifocal capsular invasion: a case report
Source: Front Oncol. 2026 Jul 7;16:1895275. doi: 10.3389/fonc.2026.1895275 (PMC13384886; doi:10.3389/fonc.2026.1895275)

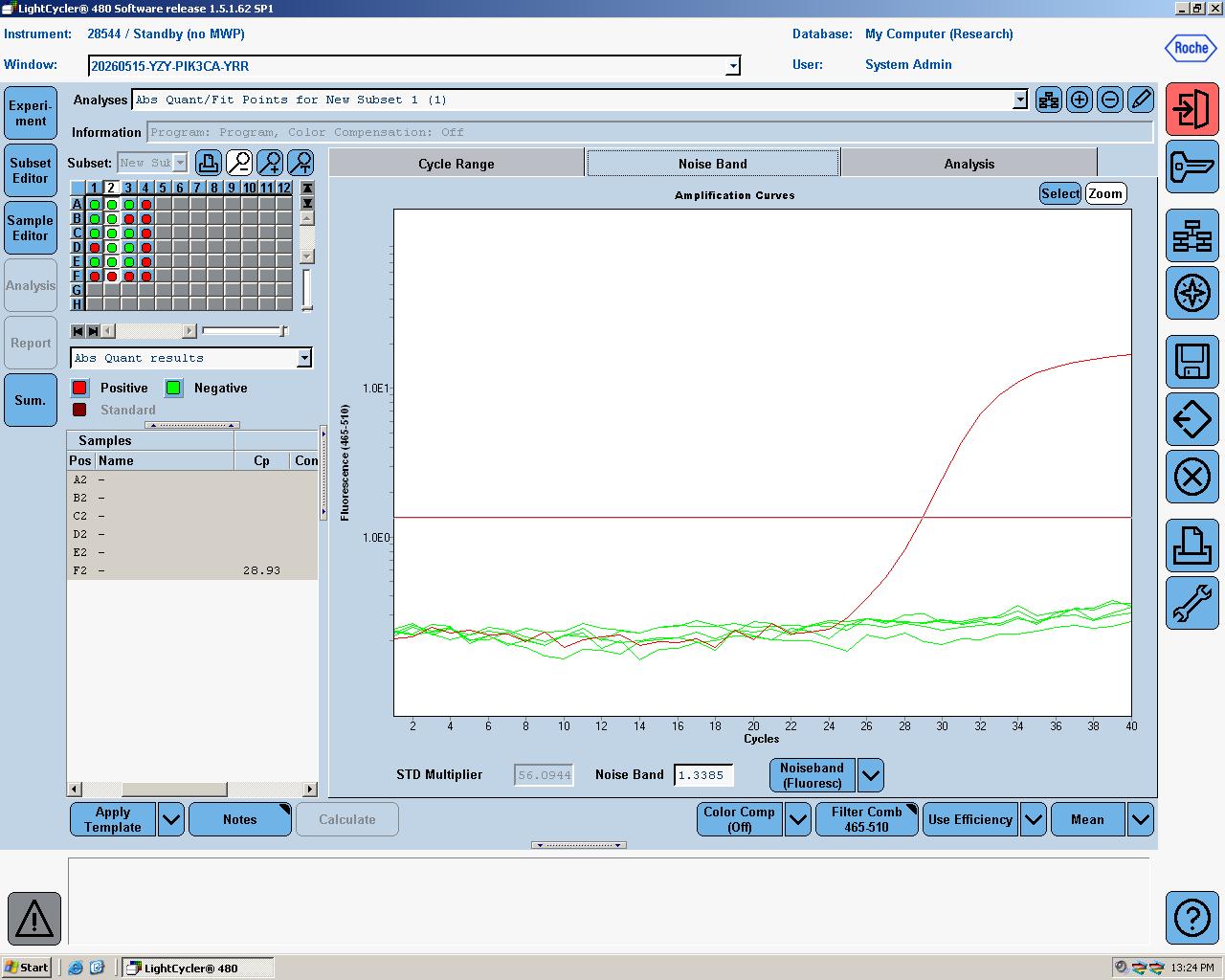

Supplement: Supplementary file 1 [file Image1.jpeg]

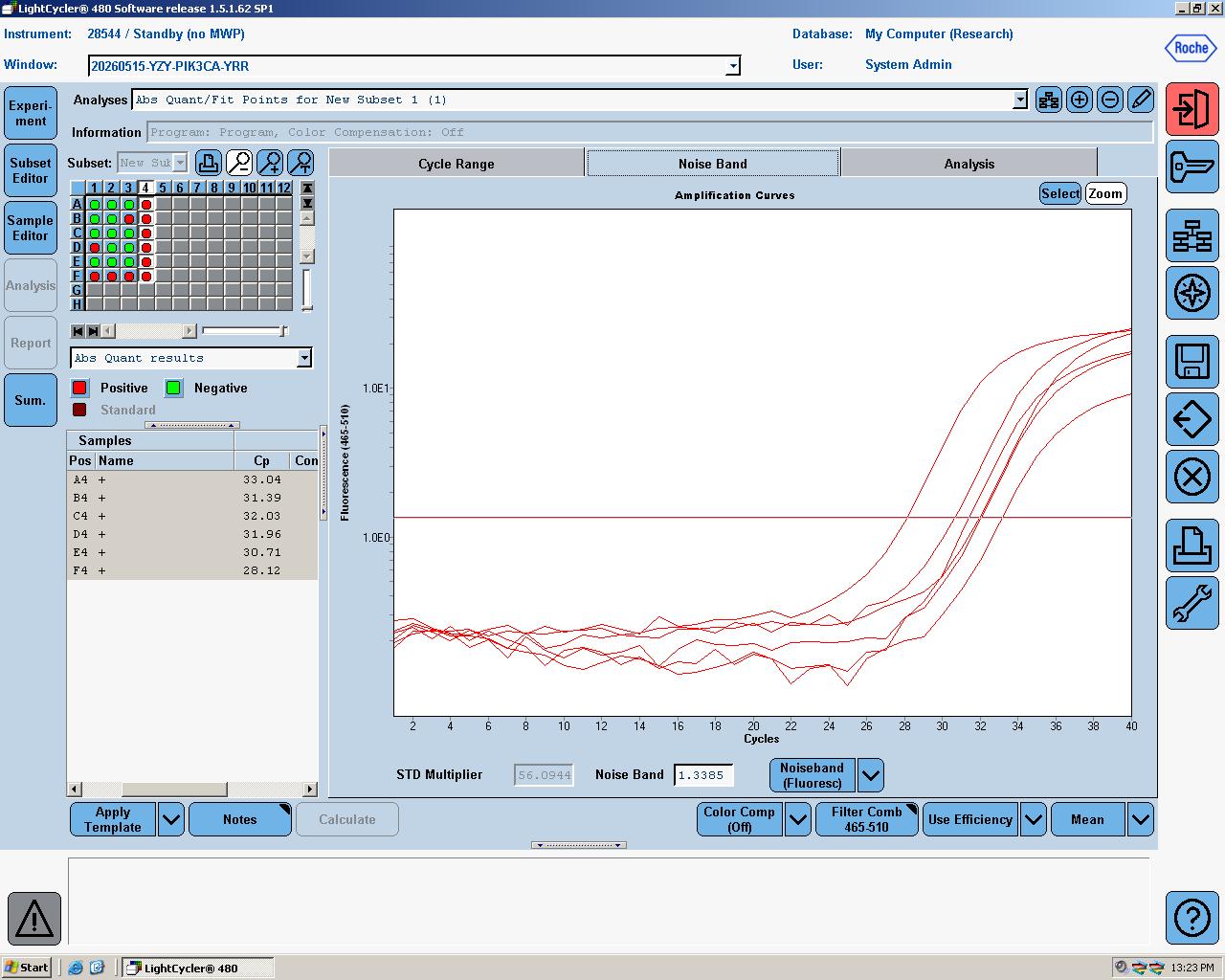

Supplement: Supplementary file 2 [file Image2.jpeg]

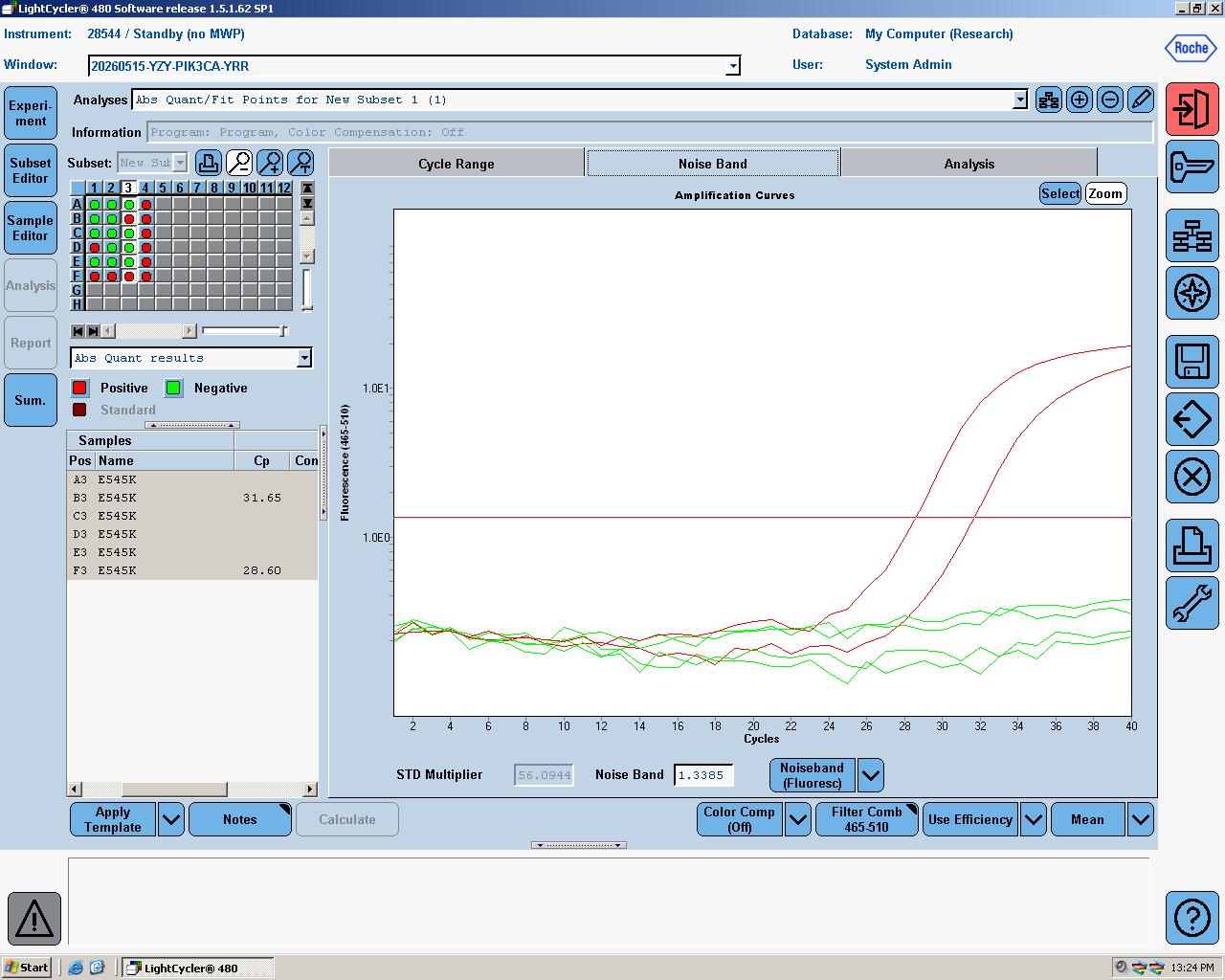

Supplement: Supplementary file 3 [file Image3.jpeg]

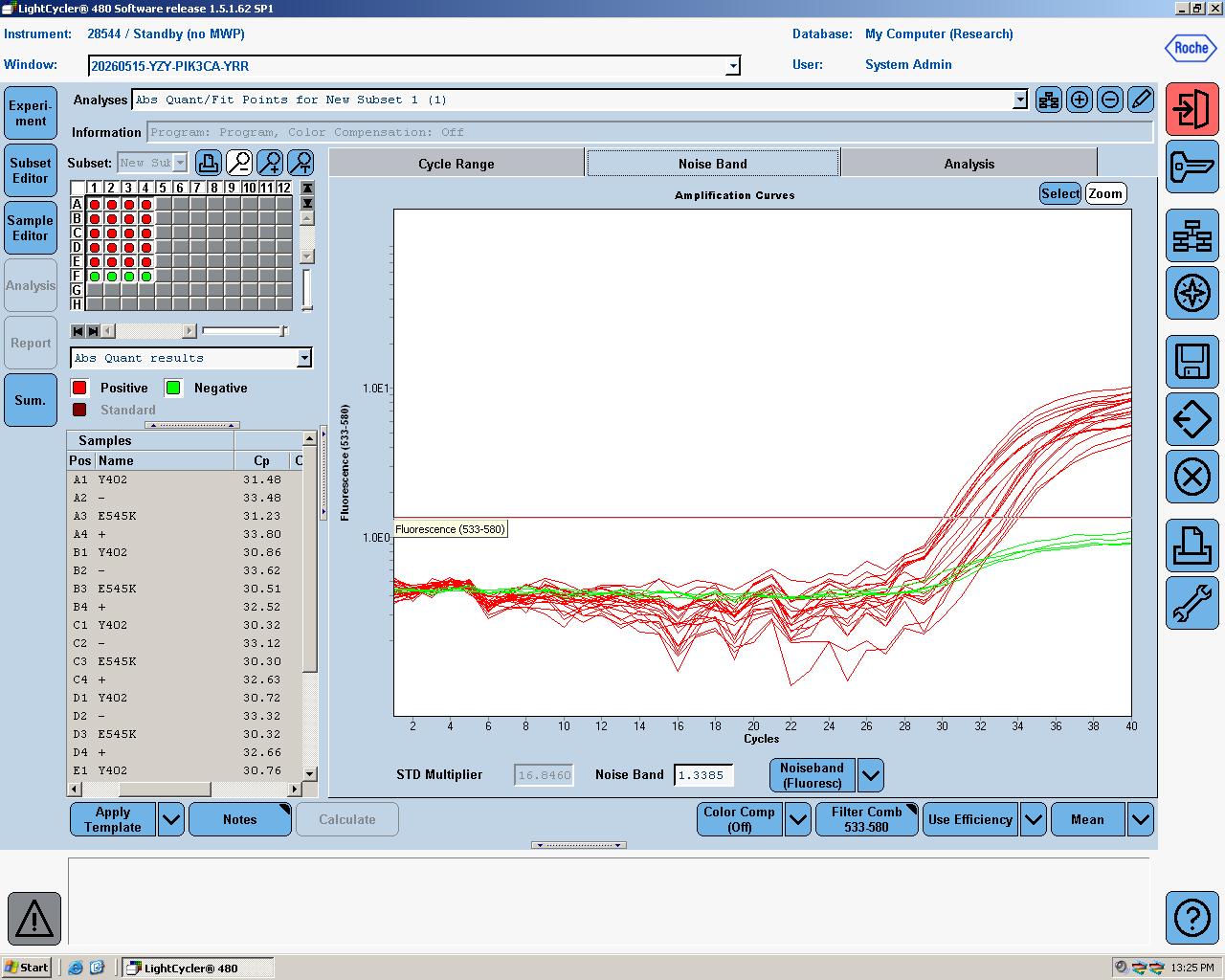

Supplement: Supplementary file 4 [file Image4.jpeg]

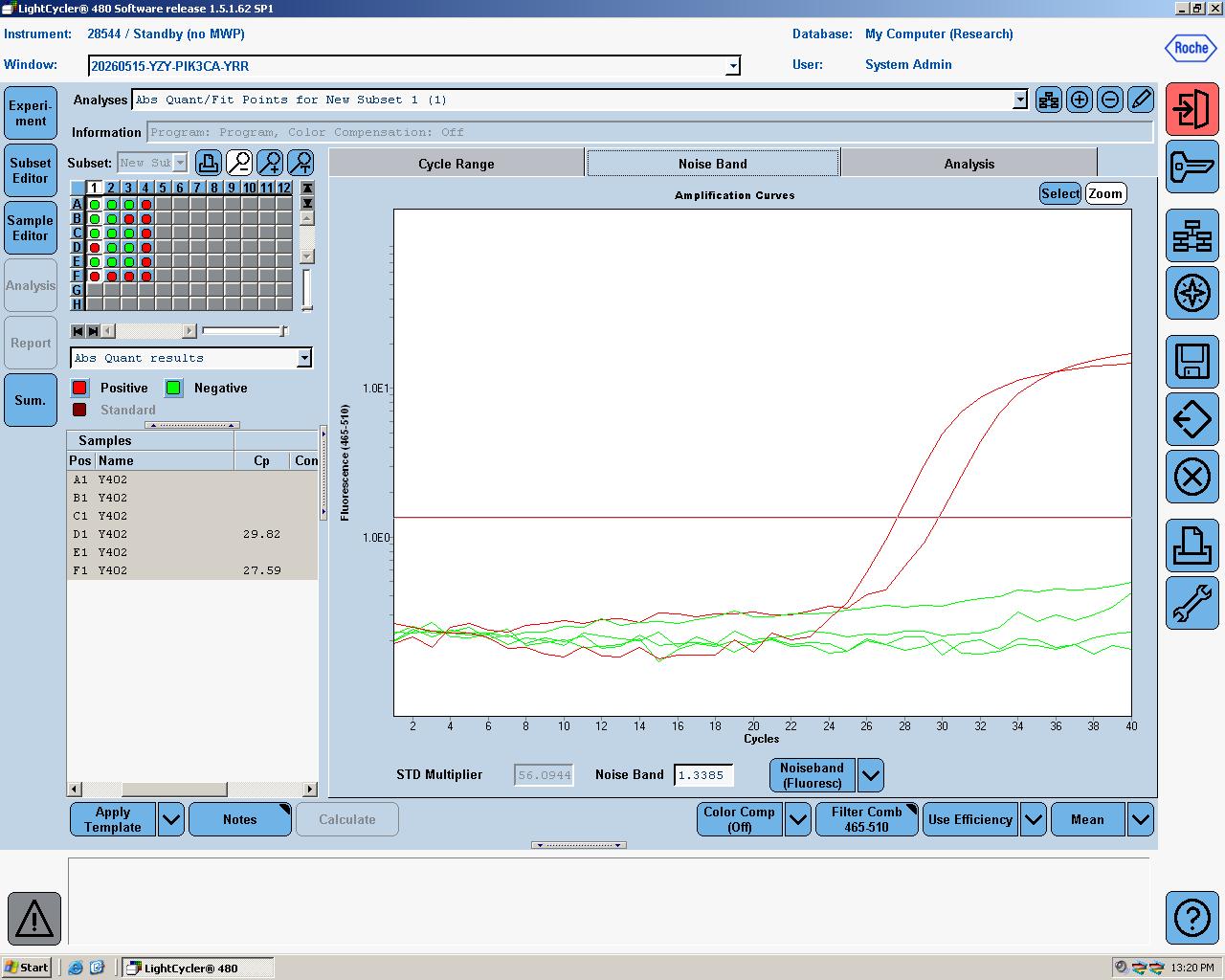

Supplement: Supplementary file 5 [file Image5.jpeg]
